# Recent advances (2022–2024) in eye-tracking for Parkinson’s disease: a promising tool for diagnosing and monitoring symptoms

**DOI:** 10.3389/fnagi.2025.1534073

**Published:** 2025-05-21

**Authors:** Laura Culicetto, Davide Cardile, Giulia Marafioti, Viviana Lo Buono, Francesca Ferraioli, Simona Massimino, Giuseppe Di Lorenzo, Chiara Sorbera, Amelia Brigandì, Carmelo Mario Vicario, Angelo Quartarone, Silvia Marino

**Affiliations:** ^1^IRCCS Centro Neurolesi “Bonino-Pulejo”, Messina, Italy; ^2^Dipartimento di Scienze Cognitive, Psicologiche, Pedagogiche e Degli Studi Culturali, Università Degli Studi di Messina, Messina, Italy

**Keywords:** eye tracking, Parkinson’s disease, assessment, machine learning, virtual reality

## Abstract

**Introduction:**

Parkinson’s disease (PD) is one of the most prevalent neurodegenerative disorders, characterized by both motor and non-motor symptoms, including impaired oculomotor functions. Eye-tracking technology, a precise and non-invasive method for measuring eye movements, has emerged as a promising tool for diagnosing and monitoring PD progression. This systematic review evaluates the effectiveness of eye-tracking in assessing motor and cognitive alterations associated with PD.

**Methods:**

A systematic review of the literature was conducted using PubMed, Web of Science, Embase, Scopus and Cochrane Library databases to identify studies applying eye-tracking to assess oculomotor functions in PD patients. Only articles published from 2022 to 2024 were considered.

**Results:**

A total of 10809 studies were identified. 18 met the inclusion criteria and were included. Findings indicate that eye-tracking may offer valuable insights into both oculomotor and cognitive dysfunctions. Specific metrics such as saccade velocity, fixation duration, and pupil size have been correlated with disease severity. Recent technological advancements, including the integration of machine learning (ML) and virtual reality (VR), have further enhanced the diagnostic accuracy and scalability of eye-tracking methods.

**Conclusion:**

In the past 3 years, eye-tracking has rapidly advanced, particularly through its integration with ML and VR. These innovations have enhanced precision, accessibility, and clinical relevance. Emerging evidence also supports its potential to detect eye movement biomarkers associated with disease stage, motor subtypes, and cognitive decline. This review synthesizes the latest findings, underscoring the role of eye-tracking as a scalable and personalized tool for PD assessment. However, further efforts are needed to address challenges such as protocol standardization and device variability.

**Systematic review registration:**

https://www.crd.york.ac.uk/prospero/display_record.php?ID=CRD42024602802, identifier CRD42024602802.

## 1 Introduction

Parkinson’s disease (PD) is a progressive neurodegenerative disorder, caused by the loss of dopaminergic neurons in the basal ganglia. It is currently diagnosed through clinical evaluation of motor symptoms, including tremor, rigidity, akinesia, and postural instability, as well as non-motor symptoms such as sleep disturbance, cognitive impairment and mood disorders (Bloem et al., 2021; Chaudhuri et al., 2006; Culicetto et al., 2024; Jankovic, 2008; Pennisi et al., 2023).

Its incidence is 10–18 cases per 100,000 person-years (de Lau and Breteler, 2006). The key risk factors include gender, with men affected more frequently than women at a ratio of approximately 3:2 (de Lau and Breteler, 2006). Ethnicity also plays a role, with the highest incidence reported among Hispanic individuals in the USA, followed by non-Hispanic Whites, Asians, and Black individuals (Van Den Eeden et al., 2003). However, age is the strongest risk factor, for developing PD. Approximately 83% of PD patients develop Parkinson’s Disease Dementia (PDD), linking the condition to aging (Hely et al., 2008) and emphasizing the need for early, accurate diagnosis to optimize treatment and outcomes (Braak et al., 2003; Irwin et al., 2013).

Impaired oculomotor behaviors, such as smooth pursuit movements and saccades, are observed in approximately 75% of PD patients, making them a potential clinical indicator (Corin et al., 1972; Fooken et al., 2022; Helmchen et al., 2012). The neural pathways controlling eye movements, spanning from the frontal cortex to the medulla, reflect brain circuit integrity (Coe and Munoz, 2017). In PD, neurodegeneration disrupts these circuits, leading to increased saccade latency, antisaccade errors, saccade hypometria, altered pupil responses, and decreased blink rates. These changes worsen with disease progression and may precede motor symptoms, offering utility for early diagnosis and disease monitoring (Antoniades and Kennard, 2015; Crawford et al., 1989; Perkins et al., 2021).

Research suggests a link between motor symptoms and eye movement disorders in PD. Eye movement impairment can exacerbate motor symptoms, such as reduced eye-hand coordination, leading to delay movements and decreased overall coordination (Terao et al., 2017).

While dopamine replacement therapy and deep brain stimulation (DBS) manage motor symptoms (Hartmann et al., 2019), treatment for non-motor symptoms, including visual impairments, remain limited. Some PD treatments, like DBS, improve eye movements (Antoniades et al., 2014; Fawcett et al., 2010; Nilsson et al., 2013; Yugeta et al., 2010), while others may cause visual side effects, such as hallucinations (Armstrong, 2011).

Currently, two methods are used to assess eye movements in patients. The first one involves direct observation, where patients follow the physician’s finger to detect abnormal eye movements. The second utilizes eye-tracking devices for more accurate monitoring. However, the direct observation method has notable limitations. Physicians can only assess visual responses based on finger movements, and the method’s low resolution and reliance on subjective perception make it challenging to detect subtle abnormalities that might otherwise go unnoticed (Rosengrant et al., 2021).

Eye-tracking is a technique used to record eye movements and gaze position over time during various tasks. It is commonly employed to study the distribution of visual attention (Carter and Luke, 2020). Eye-tracking technology, especially with advancements in non-invasive infrared systems, has shown great clinical potential as a non-invasive and objective marker. However, temporal and spatial resolution can vary across different systems, and not all eye-tracking devices offer the same precision. Although the underlying neural mechanisms still remain unclear, oculomotor dysfunctions in PD reflect complex brain changes, potentially making eye-tracking useful instrument to monitoring disease’s progression (Pelzer et al., 2020; Perneczky et al., 2011; Yugeta et al., 2013).

Motor impairments in PD are mirrored by oculomotor abnormalities such as saccade hypometria (Terao et al., 2013; White et al., 1983), delayed initiation of voluntary saccades (Terao et al., 2011), multistep saccades (Blekher et al., 2009), and impaired smooth pursuit (Almer et al., 2012; Fukushima et al., 2017; Shibasaki et al., 1979). Voluntary eye movements, particularly memory-guided and antisaccades, are more severely affected due to combined superior colliculus inhibition and cortical dysfunction (Terao et al., 2011; Terao et al., 2013). As the disease progresses, reflexive saccades also become hypometric and delayed (Gorges et al., 2014; Terao et al., 2011).

Non-motor symptoms are likewise reflected in eye movement patterns. Increased antisaccade errors and prolonged antisaccade latencies are associated with executive dysfunction, impaired inhibitory control from the dorsolateral prefrontal cortex (DLPFC), and basal ganglia dysfunction (Crawford et al., 2002; Terao et al., 2013; van Stockum et al., 2008). These deficits have been linked to clinical features such as freezing of gait (FOG) and impaired postural control (Ewenczyk et al., 2017; Walton et al., 2015).

Eye-tracking, therefore, provides a non-invasive and objective method for capturing both motor and non-motor dysfunctions in PD, offering potential biomarkers for early diagnosis, disease staging, and monitoring of progression.

Additionally, research has shown that other neurodegenerative diseases, including corticobasal syndrome, progressive supranuclear palsy (PSP), and multiple system atrophy, exhibit distinct patterns of eye movement abnormalities, indicating that eye-tracking could be useful in the differential diagnosis of Parkinson-plus syndromes (Baird-Gunning and Lueck, 2018; Henderson et al., 2011).

Previous reviews, such as Waldthaler et al. (2021), comprehensively synthesized the application of eye-tracking in PD up to 2021, focusing primarily on diagnostic potentials based on oculomotor dysfunctions. Their findings highlighted the role of antisaccade errors, saccade hypometria, and fixation instability as early markers of PD and cognitive decline. Specifically, Waldthaler et al. (2021) conducted a comprehensive meta-analysis on antisaccade performance in PD, confirming that patients exhibit significantly higher antisaccade error rates and prolonged antisaccade latencies compared to healthy controls (HCs). They also demonstrated that motor disease severity, indexed by disease duration, Hoehn & Yahr stage, and UPDRS III scores, was positively correlated with increased antisaccade latency. Notably, acute levodopa administration did not significantly affect antisaccade performance, whereas subthalamic nucleus deep brain stimulation (STN-DBS) appeared to decrease antisaccade latency, possibly reflecting a shift toward greater motor impulsivity. Waldthaler et al. (2021) emphasized the potential of antisaccade measures as markers for disease severity, but also highlighted the need for longitudinal studies to validate their prognostic value.

Building upon these earlier findings, the present review aims to underscore the growing utility of eye-tracking technology in the diagnosis and monitoring of PD, with a particular focus on recent technological advancements. To avoid redundancy and to emphasize the significant progress made, including the integration of machine learning (ML) algorithms and virtual reality (VR)-based protocols, we concentrated on studies published between 2022 and 2024. For a more detailed historical overview, we reccomend referring to Waldthaler et al. (2021).

## 2 Materials and methods

**FIGURE 1:**
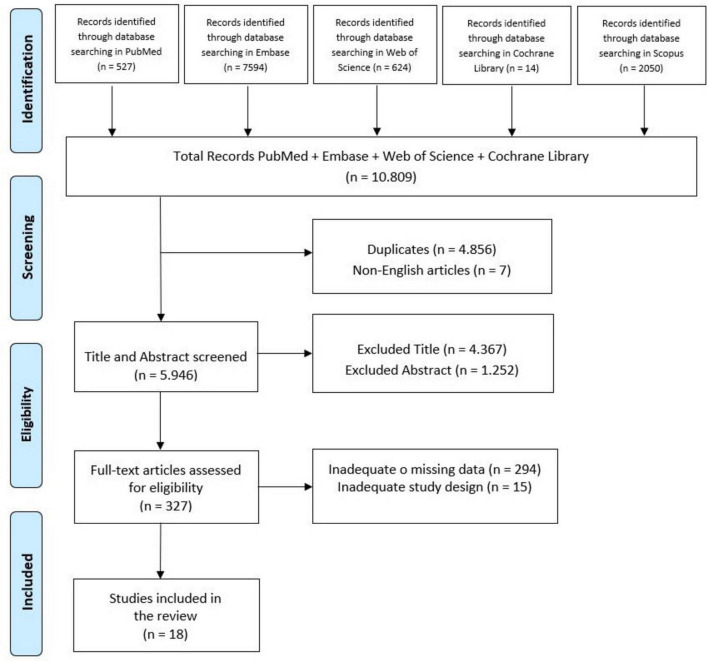
Search and selection of eligible articles.

This systematic review was conducted and reported in compliance with the Preferred Reporting Items for Systematic Review and Meta-Analyses (PRISMA) guidelines (Page et al., 2020; Figure 1). The review protocol was registered on PROSPERO with the registration number CRD42024602802.

### 2.1 PICO model

We utilized the PICO framework (Population, Intervention, Comparison, and Outcome) to define our research question. The target population includes adults (>18 years) with PD. The intervention involves eye-tracking technology, with comparisons drawn against healthy individuals or those with other neurodegenerative disorders. The outcome focuses on the effectiveness of eye tracking in enhancing the accuracy of diagnosing and monitoring PD progression. This research investigates the potential of eye tracking in ’assisting physicians in the detection and management of PD.

### 2.2 Search strategy

Studies were identified through a database search of PubMed, Web of Science, Embase and Scopus databases in September 2024. Articles meeting the predefined inclusion criteria were evaluated for potential inclusion. The search strategy employed the following string: (Parkinson[Title/Abstract]) AND ((“eye tracking”[Title/Abstract]) OR (eye-tracking[Title/Abstract]) OR (eyetracking[Title/Abstract]) OR (video oculography[Title/Abstract]) OR (video-oculography [Title/Abstract]) OR (videooculography[Title/Abstract]) OR (elec tro oculography[Title/Abstract]) OR (electro-oculography [Title/Abstract]) OR (electrooculography[Title/Abstract]) OR (eye movement[Title/Abstract]) OR (eye-movement[Title/Abstract]) OR (eyemovement[Title/Abstract])). Search terms targeted titles and abstracts. After removing duplicates, all remaining articles were screened based on title and abstract. Only studies published between 2022 and 2024 were included in the review. As part of the supplementary search strategy, we performed backward citation tracking by screening the reference lists of all included articles to identify any additional studies that met the inclusion criteria but were not captured through the initial database search. However, this additional step did not yield further studies that met our inclusion criteria.

A study was included if it described or investigated oculomotor function in patients with PD. Only studies conducted on human populations and published in English that met the following criteria were included: (i) primary empirical studies employing observational (cross-sectional, cohort, and case-control), experimental (e.g., randomized controlled trials), interventional (e.g., pharmacological trials), feasibility designs; (ii) articles that employed eye-tracking technology to assess oculomotor function.

A study was excluded if there was a lack of data or information about eye-tracking technology in patients with PD. Additionally, in accordance with PRISMA 2020 guidelines (Page et al., 2020), we excluded non-primary literature (e.g., systematic reviews, meta-analyses), conference abstracts or proceedings, editorials, letters, books, and single-case studies. These thresholds were adopted to ensure methodological rigor, data transparency, and to reduce the potential for bias due to insufficient reporting or limited generalizability.

### 2.3 Study selection

To minimize bias and ensure a rigorous selection process, two authors (L.C. and D.C.) independently reviewed and extracted data from the studies. Any discrepancies were resolved through collaborative discussion, with consultation from a third author (V.L.B). This multi-step approach ensured that at least three researchers independently assessed each article. In cases of persistent disagreement, the final decision involved all authors.

### 2.4 Data extraction and analysis

The studies that met the inclusion criteria were summarized based on the following points: (Bloem et al., 2021) Study characteristics, including the type of study and the country where the data were collected; (Chaudhuri et al., 2006) Patients characteristics, such as the sample size, age, gender, duration of disease, and the level of education; (Culicetto et al., 2024) Instruments utilized for eye tracking; and (Jankovic, 2008) key findings and relevant outcomes.

Following the full-text selection, data were extracted from the included studies and organized in a table using Microsoft Excel (Version 2021). The extracted data included: study title, first author name, year of publication, study aims and design, sample size, type of participants, type of intervention and control, baseline performance, type of outcome and time-points for assessment, results, and key conclusions.

Additionally, the inter-rater agreement between the two reviewers (L.C. and D.C.) was assessed using kappa statistics. A kappa score above 0.61 was set as threshold for substantial agreement, indicating strong concordance between the reviewers. This criterion ensures a rigorous assessment of inter-rater reliability, emphasizing the substantial level of agreement achieved during the data extraction process.

### 2.5 Risk of bias within individual studies

**FIGURE 2:**
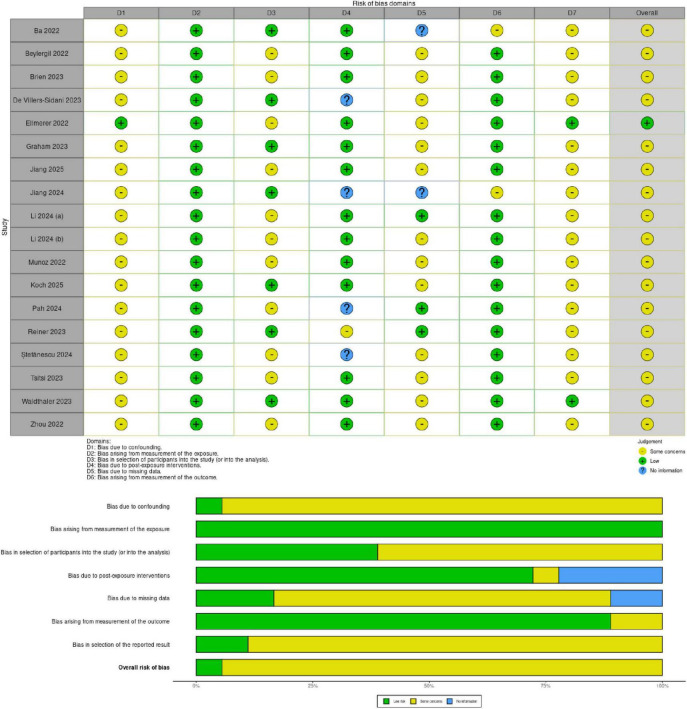
Shows the risk of bias (ROBINS-E) of studies regarding the use of eye-tracking in PD.

The risk of bias in the selected studies was independently assessed by L.C. and D.C. using the revised Cochrane tool for non-randomized controlled studies-of exposures (ROBINS-E) tool (Figure 2), which comprises seven domains: (i) bias due to confounding, (ii) bias arising from measurement of the exposure, (iii) bias in selection of participants into the study (or into the analysis), (iv) bias due to post-exposure interventions, (v) bias due to missing data, (vi) bias arising from measurement of the outcome, (vii) bias in selection of reported result.

## 3 Results

### 3.1 Synthesis of evidence

A total of 10,809 articles were identified through database searches. After removing duplicates, 4,855 studies were screened based on their title and abstract. Following full text selection, 18 studies were included for analysis. The selection process is illustrated in Figure 1. The main features of included studies were summarized in Table 1.

**TABLE 1:** Main characteristics of the included studies.

Study	Study design	Population	Hoehn & Yahr stage	Years since diagnosis/disease duration	Dopaminergic Medication Use	Instrument	Cognitive, motor, or affective tests	Outcome
Ba et al., 2022	Observational study	20 PD (66.8 ± 8.4); 24 HC (62.2 ± 7.5)	1 to 4, with 45% in stage H&Y ≥ III	Disease course of 9.8 ± 4.7 years	LEDD: 1219.7 ± 719.9 mg. On medication state	Tobii X2-60 eye tracker, sampling rate 60 Hz	MoCA	Patients with PD exhibited significant stereopsis deficits, longer visual response times, and less accurate saccadic movements. These deficits correlated with UPDRS-III and MoCA
Beylergil et al., 2022	Cross-sectional	3 PD (1 female, 67 ± 9.2 years); 7 HC (3 females; 64.29 ± 7.54)	2–4	Disease duration of 11.38 ± 5.39 years	Stable dosage of antiparkinsonian medication for at least 6 months before participation. On medication state.	EyeLink 1000 (SR Research, Canada), 500 Hz resolution	MDS-UPDRS-III	PD patients showed increased fixational saccades, reduced exploratory saccades, and delayed target detection, especially in unexpected locations, indicating visuospatial deficits
Brien et al., 2023	Observational, longitudinal cohort design	121 (29 females; 67.9 years ± 6.5) 55 classified as cognitively normal, 45 with MCI, and 20 with dementia (PDD); 106 HC (67.7 ± 8.2).	1–3	Participants diagnosed within the last 3–8 years	LEDD: 692.2 ± _365.1 mg No information on ON/OFF medication state.	Video-based eye-tracking, sampling rate not reported	MoCA; MDS-UPDRS; SCOPA-AUT, NPSY	Interleaved pro/antisaccade task showed altered saccade latency, error rates, pupil responses, and blink behavior in PD. A ML classifier (AUC = 0.88, 83% sensitivity, 78% specificity) correlated with MDS-UPDRS and MoCA scores, supporting eye-tracking as a biomarker for PD diagnosis and progression.
de Villers-Sidani et al., 2023	Cross sectional study	59 PD (63.76 ± 8.23); 62 HC (56.64 ± 8.56)	NA	NA	NA No information on ON/OFF medication state.	12.9-inch iPad Pro tablet with the ETNA software. Sampling rate 60 Hz	Motor scale of MDS-UPDRS	Tablet-based eye-tracking assessed fixation stability, prosaccades, and antisaccades in PD, revealing increased saccadic intrusions, prolonged latencies, reduced accuracy, and higher error rates, supporting its potential as a biomarker for monitoring PD and distinguishing patients from HC.
Ellmerer et al., 2022	Phase II, randomized, placebo-controlled, double-blind, parallel-group pilot study	47 PD patients: Nabilone group: 65.9 ± 7.5 years, Placebo group: 62.9 ± 9.3 years	NA	Disease duration: -Nabilone group: 7.0 ± 5.7 years -Placebo group: 5.4 ± 2.0 years	No information on ON/OFF medication state	Tobii TX-300, sampling rate 300 Hz	MoCA, MMSE, MDS-UPDRS, HADS	Prosaccades, antisaccades, Go/NoGo, and mixed pro-/antisaccade tasks showed no significant difference in reaction times or error rates between the nabilone and placebo groups.
Graham et al., 2023	Interventional study	43 PD (68.86 ± 6.56) 17 HC (69.24 ± 7.97)	2–3	Mean disease duration of 7.77 years (SD 5.73)	LEDD: 817.46 mg (SD 409.61) for all PD participants. On medication state.	Tobii Pro Glasses 2, sampling rate 100 Hz	MDS-UPDRS III, MoCA, Clock Drawing Test,	Visual cues improved gait (stride length, stride time variability) and increased saccade frequency but reduced saccade amplitude and peak velocity. PD patients showed reduced saccade frequency and prolonged fixations, with FOG patients exhibiting more severe impairments.
Li et al., 2024a	Observational cross-sectional study	145 PD, mean age 65 years; 80 HC	1.5	Average disease duration: 3 years	All participants were assessed in the ON-medication state	Eyeknow eye tracker, sampling rate 120 Hz	MoCA; MMSE; UPDRS	PD patients exhibited ocular tremor independent of disease severity, along with fixation inaccuracy, slower saccades, impaired visual search, and altered gait parameters.
Li et al., 2024b	Cross-sectional observational study	127 PD (63.8 ± 8.4) 80 HCs (63.8 ± 8.4)	51% of PD patients had H&Y stage ≥ 2	Disease duration: 3.0 years	LEDD: 237.5 mg (median, with interquartile range 0.0–476.9 mg). ON-medication state	EyeKnow system (infrared pupil and corneal reflection tracking, 120-Hz sampling rate)	UPDRS, FOGQ, MoCA, MMSE, HAM-D, Sleep Scale 2, Epworth Sleepiness Scale, REM Sleep Behavior Disorder Screening Questionnaire	PD patients showed fixation inaccuracy (fixation task), delayed saccades (prosaccade/antisaccade tasks), impaired smooth pursuit (smooth pursuit task), and reduced visual search ability (visual search task), correlating with disease stage and motor subtypes.
Jiang et al., 2024	Observational study – Cross sectional	44 PD; 22 HC	1–3	Average disease duration was 1.66 ± 1.98 years?	NA No information on ON/OFF medication state.	HMD device (HTC Vive Pro eye) with precise eye movement tracking technology. Sampling rate 120 Hz	NA	PD patients showed slower saccades, higher error rates, and impaired visual scanning with prolonged fixation duration and fewer fixations. A VR-based eye-tracking model (SVM classifier) achieved 83.4% accuracy, supporting its potential for early PD diagnosis.
Jiang et al., 2025	Cross-sectional study	14 PD (66.95 ± 8.84) years 125 HC (63.64 ± 6.9)	NA	Mean disease duration of 1.66 ± 1.98 years	NA No information on ON/OFF medication state.	HTC Vive Pro Eye (virtual reality-based system). Sampling rate 120 Hz	UPDRS III	VR-based tasks (gaze stability, prosaccades, antisaccades, smooth pursuit) showed impaired fixation, saccades, and pursuit in PD; deep learning model achieved 92.73% accuracy for diagnosis.
Koch et al., 2024	Cross sectional study	65 PD (64.14 ± 8.40)	2.15 ± 0.69 (Range: 1–4)	NA	ON state, with a stable dosage of antiparkinsonian medication	Tablet-based eye-tracking system (ETNA, Innodem Neurosciences), sampling rate 60 Hz	UPDRS-III, MoCA, TMT, COWAT-CFL, HVLT	Oculomotor tasks (fixation, prosaccades, antisaccades, smooth pursuit, and optokinetic nystagmus) correlated with disease severity and cognitive scores. A ML model classified mild vs. moderate PD with 90% accuracy using oculomotor parameters.
Munoz et al., 2022	Within-subject crossover study	33 PD (66.27 ± 4.86 years) HC 13 (65.23 ± 5.72 years?)	2.15 ± 0.36	Mean disease duration = 6.85 ± 4.07 years.	LEDD = 755.15 ± 636.03 mg; participants were tested both OFF and ON medication	Eye-tracking system (Eyelink II, SR Research Ltd), sampling rate 500 Hz	MoCA, UPDRS-III,	Dopaminergic medication prolonged saccade latency and reduced gain and peak velocity across gap, step, and overlap tasks, with the strongest impairments in the overlap task, particularly in peak velocity and gain.
Pah et al., 2024	Case-control observational study	42 PD patients (70.5 ± 10.4 years) 28 healthy controls (69.7 ± 7.6 years)	NA	Mean disease duration was 3.9 ± 3.5 years; disease duration of 5 years or less.	NA ON-medication state	GP3 Eye Tracker, sampling rate 60 Hz	NA	PD patients exhibited shorter saccadic latencies, increased inaccuracy in target reaching (saccadic hypometria), and greater gaze instability, suggesting reflexive saccadic alterations as potential PD biomarkers.
Reiner et al., 2023	Cross-sectional study	215 PD 79 females 69 ± 9.1 years	Mild-to-moderate PD: H&Y ≤ 2 Severe PD: H&Y > 3	NA	159 out of 215 PD patients (73.9%) were under levodopa medication treatment. The ON/OFF state during testing is not specified.	Tobii Fusion Pro, Sampling rate not reported	MDS-UPDRS III	PD patients showed longer saccadic latency, increased antisaccade errors, and reduced accuracy, worsening with disease severity.
ŞtefŞnescu et al., 2024	Retrospective study	62 (60.35 ± 8.98 years)	2–3	NA	NA No information on ON/OFF medication state.	Tobii TX300 eye-tracking system, sampling rate 250 Hz	MMSE; CANTAB; PRM; SWM	Longer saccadic latencies, reduced velocity (VGS task), and cognitive-related impairments, with blink rate and saccade duration correlating with visuospatial memory performance.
Tsitsi et al., 2023	Cross-sectional observational study	48 PD patients (64.5 ± 11.5) 42 HCs (62.5 ± 16.25)	1–3	Years since diagnosis: 2.5 (3.5) years.	LEDD reported (545 ± 496.25 mg), ON state. ON-medication state	Tobii Pro Spectrum, sampling rate 1200 Hz	UPDRS, MoCA	PD patients had fewer fixations per second and prolonged fixation duration (reading task), with deficits only in cognitively impaired PD patients (MoCA < 26). Fixation duration correlated with MoCA scores, suggesting eye-tracking as a tool for early cognitive decline detection in PD.
Waldthaler et al., 2023	Cross-sectional study	61 PD (14 females, 63.5 ± 8.5); 25 HC (8 females, 62.9 ± 11.0)	2.5	Mean disease duration across clusters: Cluster 1: 7.2 years (SD: 5.6) Cluster 2: 8.4 years (SD: 6.2) Cluster 3: 9.3 years (SD: 5.6)	LEDD: Cluster 1: 705.5 mg (SD: 411.6) Cluster 2: 660.7 mg (SD: 430.8) Cluster 3: 743.5 mg (SD: 342.3) Dopamine agonist LEDD: Cluster 1: 123.5 mg (SD: 104.6) Cluster 2: 212.8 mg (SD: 224.5) Cluster 3: 147.9 mg (SD: 143.5) All participants were assessed in the ON-medication state.	EyeLink 1000 Plus, sampling rate not reported	MoCA, TMT, RCF, FAB, BDI, MDS-UPDRS III.	Prosaccades and antisaccades (horizontal/vertical) tasks revealed vertical saccadic hypometria, suggesting a potential PD biomarker.
Zhou et al., 2022	Cross-sectional study	75 *de novo* (PD) (64.5 ± 8.1) 75 patients with ET 46 HCs	50.7% of PD patients were at stage ≥ 2	Disease duration: s 12 months (6–24 months)	Drug-naïve *de novo* PD patients	Visual Eyes 4-channel videonystagmography (Micromedical Technologies, USA), sampling rate 120 Hz	MMSE, MoCA, NMSQuest, RBDSQ REM, NMSQuest, HAM-D	PD patients showed longer saccadic latency (visually guided saccades) and reduced smooth pursuit gain (SPEM), correlating with disease severity. Eye-tracking differentiated PD from controls (AUC = 0.78) but not from ET.

HC, healthy control; MoCA, Montreal Cognitive Assessment Test; MMSE, Mini–Mental State Examination; UPDRS, Unified Parkinson’s Disease Rating Scale; NA, not available; ADFES, Amsterdam Dynamic Facial Expression Set; MCI, with mild cognitive impairment; MDS-UPDRS, Society-Unified Parkinson’s Disease Rating Score; SCOPA-AUT, Scales for outcomes in Parkinson’s disease- Autonomic Dysfunction; NPSY, neuropsychology battery; SVM, Support Vector Machine; POM, pursuit ocular movements; H&Y, Hoehn & Yahr; CANTAB, Cambridge Neuropsychological Test Automated Battery; PRM, Pattern Recognition Memory; SWM, Spatial Working Memory; ML, Machine learning; FAB, Frontal Assessment Battery; BDI, Beck Depression Inventory-II (BDI-II); OMO, Odd Man Out; EOG, electrooculography; TMT, Trail Making Test; HVLT, Hopkins Verbal Learning Test; COWAT-CFL, Controlled Oral Word Association Test; HADS, Hospital Anxiety and Depression Scale; GDS-15, Geriatric Depression Scale; FOGQ, freezing of gait questionnaire; HAM-D, Hamilton Depression and Anxiety Scales; RBDSQ, REM Sleep Behavior Disorder Screening Questionnaire; NMSQuest, Non-Motor Symptoms Questionnaire; RBDSQ REM Behavior Disorder Screening Questionnaire; ET, essential tremor; ML, machine learning; VGS, visually-guided saccades; LEDD, Levodopa Equivalent Daily Dosage.

### 3.2 Key findings from included studies

#### 3.2.1 Diagnostic application of eye-tracking

The studies included in this review highlight the use of eye-tracking technology as a promising non-invasive tool for assessing motor and cognitive functions in PD patients. Several studies have demonstrated the potential for eye-tracking’s diagnostic capabilities, distinguishing PD patients from HCs based on metrics related to saccadic movements, fixation stability, and other oculomotor functions. Waldthaler et al. (2023) analyzed eye-tracking data from 61 PD patients and 25 HCs and they identified three distinct patterns of saccade impairment in PD. The findings revealed: increased express saccades and anti saccade errors, associated with executive dysfunction; delayed and hypometric saccades, linked to multidomain cognitive decline and longer antisaccade latencies but preserved accuracy, with no cognitive impairment. A common feature across all groups was vertical saccadic hypometria, reinforcing its potential as a biomarker for PD. Zhou et al. (2022) investigated oculomotor impairments in newly diagnosed, drug-naïve PD patients, exploring their potential as early biomarkers for disease detection and progression. Using video nystagmography-based eye tracking, the study compared 75 PD patients, 75 essential tremor (ET) patients, and 46HCs, assessing saccadic latency, saccadic accuracy, and smooth pursuit eye movement (SPEM) gain. The results showed that both PD and ET patients exhibited prolonged saccadic latency and reduced saccadic accuracy compared to HCs. However, PD patients displayed significantly impaired SPEM gain across all tested frequencies, whereas ET patients only showed reduced gain at 0.4 Hz. Longer saccadic latency correlated with disease duration, while lower SPEM gain was linked to greater motor severity in PD. A model incorporating eye-tracking parameters successfully differentiated PD from HCs with 80.4% sensitivity and 73.3% specificity, but it was not effective in distinguishing PD from ET.

#### 3.2.2 Eye-tracking and cognitive impairment detection

Using a high-resolution eye tracker (Tobii Pro Spectrum, 1200 Hz), Tsitsi et al. (2023) investigated reading performance differences between PD patients and HCs to assess whether these alterations stem from cognitive decline rather than oculomotor dysfunction. The study found that PD patients had longer fixation durations and fewer fixations per second than HCs. However, only cognitively impaired PD patients (Montreal Cognitive Assessment, MoCA < 26) showed prolonged fixations and slower reading speeds, while cognitively intact PD patients (MoCA ≥ 26) performed similarly to controls. Fixation duration also correlated with MoCA scores, indicating a link to cognitive status. These results support eye-tracking as a non-invasive tool for detecting early cognitive decline in PD, with fixation-based metrics offering potential for screening and disease monitoring.

#### 3.2.3 Monitoring motor symptoms and disease severity

Pah et al. (2024) found that PD patients had shorter saccadic latency but greater inaccuracy, often overshooting targets, along with increased gaze instability during fixation. While invalid saccades occurred at similar rates in PD and controls, filtering them out revealed distinct differences in reflexive saccadic behavior. These findings suggest that PD alters reflexive saccadic control, leading to faster but less precise eye movements and greater gaze instability, reinforcing eye-tracking as a promising tool for monitoring disease progression.

#### 3.2.4 Technological innovations and machine learning integration

Jiang et al. (2024) utilized VR based eye-tracking to analyze both scan paths and saccade metrics, including fixation duration, scan path length, saccade amplitude, peak velocity, latency, and error rates. Additionally, they examined the saccade-to-fixation ratio and distribution of gaze patterns, providing a comprehensive assessment of oculomotor behavior in PD patients. These features were integrated into ML models, allowing for high-accuracy classification of PD patients and highlighting the potential of VR-based eye-tracking as a novel diagnostic tool. Another recent study (Jiang et al., 2025), utilized VR-based eye-tracking and ML to assess gaze stability, pro-saccades, anti-saccades, and smooth pursuit in PD patients. The results revealed significant oculomotor impairments, including reduced gaze stability, slower and less accurate saccades, higher ant saccade error rates, and difficulties in smooth pursuit tracking. These deficits indicate motor control dysfunctions and impaired inhibitory processes, which are hallmark characteristics of PD. Similarly, de Villers-Sidani et al. (2023) employed tablet-based software (ETNA) to differentiate PD patients from controls based on basic oculomotor tasks.

Brien et al. (2023) also combined video-based eye-tracking with ML, successfully predicting motor and cognitive scores in PD, thus emphasizing eye-tracking’s potential for monitoring disease progression. The severity of PD symptoms correlates with specific eye movement abnormalities, suggesting that eye-tracking can serve as a useful marker of disease progression. Ba et al. (2022) observed impairments in fixation stability and saccadic latency, which correlated with both motor (Unified Parkinson’s Disease Rating Scale, UPDRS-III) and cognitive (MoCA) scores, further supporting eye-tracking as a marker of PD severity. Further, Li et al. (2024a) identified eye movement disorders as potential early biomarkers for PD, revealing fixation inaccuracy, delayed saccades, and impaired pursuit, linked to disease stage and motor subtypes. They developed a high-accuracy PD screening model using oculomotor parameters, cognitive scores, and education level, proposing a nomogram for clinical use, showing the diagnostic potential of eye-tracking.

Reiner et al. (2023) explored oculometric measures (OM) as biomarkers for PD severity using eye-tracking technology (Tobii Fusion Pro, Tobii, Sweden). The study analyzed eye movements in 215 PD patients and 215 HCs, correlating them with the MDS-UPDRS motor scores through computer vision and deep learning algorithms. Key findings revealed prolonged saccadic latency, higher anti-saccade error rates, and reduced response accuracy, all worsening with disease severity. Levodopa-treated patients exhibited longer saccadic latencies and higher error rates, suggesting treatment effects on oculomotor function. These results support eye-tracking as a non-invasive tool for monitoring PD progression and motor impairment, offering a potential complement to traditional clinical assessments.

Furthermore, the combined use of gait analysis and eye-tracking metrics in diagnosing PD has demonstrated increased accuracy compared to using these indicators independently (Li et al., 2024b).

Graham et al. (2023) examined visual exploration during walking in PD patients with and without FOG and HCs, assessing the impact of visual cues using mobile eye-tracking and inertial sensors. The study found that PD patients had gait impairments, worsened under dual-task conditions, while visual cues improved stride parameters and altered saccade patterns. Notably, visual exploration changes correlated with gait improvements in PD, with freezers and non-freezers responding differently. These findings suggest that visual cueing enhances both gait and visual exploration in PD, emphasizing eye-tracking as a promising tool for assessing gait impairments and cue responsiveness.

#### 3.2.5 Evaluation pharmacological effects through eye-tracking

Ellmerer et al. (2022) investigated eye-tracking as an objective tool to assess the cognitive effects of nabilone, a THC analogue used to treat non-motor symptoms in PD. This placebo-controlled pilot study found that nabilone reduced non-motor symptoms without impairing cognitive function or saccadic performance. Eye-tracking confirmed its potential as a non-invasive method for monitoring the cognitive safety of PD treatments, supporting its use in future clinical research and drug evaluation. Additionally, Munoz et al. (2022) revealed that anti-Parkinsonian medication prolongs saccade latency and reduces eye movement efficiency in PD patients, suggesting that dopaminergic therapy may impair oculomotor control despite improving motor symptoms.

#### 3.2.6 Devices and methodological variability in eye-tracking studies

A variety of eye-tracking devices were employed across the studies reviewed, ranging from stationary high-resolution systems to mobile and wearable options, resulting in variability in the findings. The studies used both video-based and electrooculography (EOG) systems, each with specific advantages and limitations. While high-precision stationary devices (e.g., EyeLink 1000, Tobii TX-300) provide superior spatial and temporal resolution, wearable options (e.g., Tobii Pro Glasses 2, HTC Vive Pro Eye, ETNA tablet-based system, EyeSeeCam, Eyelink 3, Pupil Labs Core) enable real-world tracking but may face constraints such as frame rate variability (e.g., in Tobii Glasses), which can affect data reliability for certain tasks. Koch et al. (2024) explored tablet-based eye-tracking as a biomarker for PD severity and cognitive decline. They found that prolonged saccade latency, increased anti saccade errors, and fixation instability correlated with disease progression and motor dysfunction. Eye movement impairments were also linked to executive function, attention, and memory deficits. AML model using oculomotor data achieved 90% accuracy in classifying PD severity and explained 71% of cognitive test variance. The study highlights eye-tracking as a scalable, non-invasive tool for monitoring PD progression and cognitive decline.

Beylergil et al. (2022) investigated how PD affects visual search strategies using a high-resolution video-based eye tracker. The study found that PD patients took longer to find targets, especially in unfamiliar locations, and exhibited altered eye movement patterns. They showed more fixational saccades but fewer exploratory saccades, leading to less efficient visual scanning. These findings suggest that PD-related visuomotor impairments impact attention and search efficiency, highlighting the potential of eye movement analysis as a biomarker for cognitive and motor deficits in PD.

### 3.3 Risk of bias

The Risk of Bias in Non-randomized Studies - of Exposures (ROBINS-E) tool was used to assess the risk of bias of the articles included in this review. Figure 2 shows the summary of the risk of bias assessment. Among the studies assessed, all (Ba et al., 2022; Beylergil et al., 2022; Brien et al., 2023; de Villers-Sidani et al., 2023; Graham et al., 2023; Jiang et al., 2024; Jiang et al., 2025; Koch et al., 2024; Li et al., 2024a; Li et al., 2024b; Munoz et al., 2022; Pah et al., 2024; Reiner et al., 2023; ŞtefŞnescu et al., 2024; Szymañski et al., 2017; Tsitsi et al., 2023; Waldthaler et al., 2023; Zhou et al., 2022) showed some concerns regarding the risk of bias due to confounding except for one (Ellmerer et al., 2022). Moreover, all studies showed low risk of bias arising from measurement of exposure. Further, eleven studies exhibited some concerns in the selection of participants into the study (Beylergil et al., 2022; Brien et al., 2023; Ellmerer et al., 2022; Jiang et al., 2025; Li et al., 2024a; Li et al., 2024b; Munoz et al., 2022; Pah et al., 2024; ŞtefŞnescu et al., 2024; Tsitsi et al., 2023; Zhou et al., 2022). Thirteen studies showed a low risk of bias due to post-exposure interventions (Ba et al., 2022; Beylergil et al., 2022; Brien et al., 2023; Ellmerer et al., 2022; Graham et al., 2023; Jiang et al., 2025; Koch et al., 2024; Li et al., 2024a; Li et al., 2024b; Munoz et al., 2022; Tsitsi et al., 2023; Waldthaler et al., 2023; Zhou et al., 2022). Thirteen studies reported some concerns about bias due to missing data (Beylergil et al., 2022; Brien et al., 2023; de Villers-Sidani et al., 2023; Ellmerer et al., 2022; Graham et al., 2023; Jiang et al., 2025; Koch et al., 2024; Li et al., 2024a; Munoz et al., 2022; ŞtefŞnescu et al., 2024; Tsitsi et al., 2023; Waldthaler et al., 2023; Zhou et al., 2022). In contrast, all studies selected reported a low risk of bias arising from the measurement of the outcome except for two (Ba et al., 2022; Jiang et al., 2024). Additionally, all studies reported some concerns in the selection of the reported result except two that showed low risk (Ellmerer et al., 2022; Waldthaler et al., 2023).

## 4 Discussion

### 4.1 Eye-tracking in PD: motor and non-motor symptoms correlations

The aim of this review is to analyze the potential of eye-tracking in the assessment and monitoring of PD symptoms. The studies report that eye-tracking technology has emerged as a powerful tool in diagnosing and tracking PD due to its ability to capture detailed data on oculomotor function. As oculomotor movements are controlled by several brain regions, including the cerebral cortex, basal ganglia, brain stem, and cerebellum, PD progression, which is marked by the degeneration of dopaminergic neurons in the substantia nigra pars compacta, inevitably affects these functions (Kennard and Lueck, 1989; Pah et al., 2024). Eye movement abnormalities assessed by eye-tracking, such as deficits in smooth pursuit and saccades, have been linked to early stages of PD and are correlated with disease severity and motor impairments (Zhou et al., 2022).

In line with these observations, several neurophysiological mechanisms have been proposed to explain the link between PD and oculomotor dysfunctions. The degeneration of dopaminergic neurons disrupts basal ganglia and frontal cortical circuits involved in voluntary eye movement control, particularly affecting the DLPFC and supplementary eye fields (SEF) (Gong and Zuo, 2025; Kahya et al., 2021; Tsitsi et al., 2021). These impairments manifest as hypometric saccades, antisaccade errors, and prolonged latencies, serving as sensitive markers of both motor and cognitive dysfunctions. Compared to traditional clinical evaluations, eye-tracking provides an objective, quantifiable, and non-invasive method with higher temporal resolution, enabling earlier detection of subtle oculomotor abnormalities (Gibbs et al., 2024; Tabashum et al., 2021). As PD progresses, cognitive and motor impairments become increasingly pronounced. Oculomotor metrics, including saccadic latency, fixation stability, smooth pursuit efficiency, and pupillary responses, emerge as valuable indicators of disease onset and progression (Gibbs et al., 2024). Distinctive eye movement abnormalities, such as hypometric saccades, increased square-wave jerks, and prolonged antisaccade reaction times, offer clinicians objective tools to differentiate PD from other neurodegenerative disorders, such as PSP, multiple system atrophy (MSA), and Alzheimer’s disease (Tabashum et al., 2021). These findings are consistent with previous work (Waldthaler et al., 2021), which already pointed to the diagnostic relevance of saccadic impairments and fixation abnormalities in PD. However, more recent studies, particularly those published after 2022, have expanded upon this knowledge by integrating ML and VR techniques, offering improved diagnostic precision and scalability.

Furthermore, eye-tracking has shown promise in distinguishing PD from other neurodegenerative diseases and in monitoring disease progression through ML approaches (ŞtefŞnescu et al., 2024). Recent studies have suggested that eye-tracking metrics, particularly those related to saccadic performance and fixation stability, offer diagnostic sensitivity and specificity comparable to traditional clinical evaluations (Bredemeyer et al., 2022; Kahya et al., 2021; Machine et al., 2023). However, technological variability, calibration challenges, and inter-individual differences in oculomotor parameters remain important limitations, underscoring the need for further standardization across studies.

Additionally, visual perception deficits are known to interfere with motor functions in PD, affecting patients’ navigation, mobility, and daily activities (Kim et al., 2011; Maschke et al., 2006). Clinical tests for stereoscopic vision are rarely performed despite their importance, as traditional methods like the Titmus stereotest lack sensitivity for certain oculomotor dysfunctions, such as convergence insufficiency and impaired vergence control, both of which are common in PD (Herrero-Gracia et al., 2025). More advanced methods, such as software-based 3D systems, are more effective in detecting subtle visual impairments and their association with disease severity (Ba et al., 2022). Few studies have explored the relationship between PD severity, as measured by the MDS-UPDRS motor score, and gaze or eye movement parameters. Previous research has identified correlations between motor scores and pro-saccade latency, pro-saccade gain, anti-saccade latency, and anti-saccade direction rate (Lu et al., 2019; Waldthaler et al., 2019). However, some studies, like Visser et al. (2019), found no significant correlation between saccade latency and UPDRS scores. In contrast, in De Villers-Sidani’s study (de Villers-Sidani et al., 2023), a significant correlation was observed between UPDRS motor scores and pro-saccade gain, the number of saccades required to reach the target, and the pro-saccade time-to-target parameter, particularly at large eccentricities. These findings suggest that eye movement metrics may serve as composite indicators of motor impairment in PD. In support of these findings, Reiner et al. (2023) demonstrated that oculometric measures, including saccadic latency, error rate, and response accuracy, correlate with MDS-UPDRS scores and disease severity. Their study further highlighted that PD patients exhibited prolonged saccadic latencies and increased error rates, with these impairments worsening as the disease progressed.

Beyond motor impairments, cognitive dysfunction in PD is a well-established risk factor for PDD. Studies suggest that early cognitive impairments, particularly those related to temporal lobe and cholinergic systems, are significant predictors of dementia (Wong et al., 2019). Eye-tracking metrics, such as fixation duration, have been shown to correlate with cholinergic deficits, indicating that these metrics could serve as early non-invasive markers for cognitive decline in PD patients. However, more longitudinal studies are required to confirm these correlations. In line with these findings, tablet-based eye-tracking technology has been shown to effectively assess both cognitive function and disease severity in PD (Koch et al., 2024). Research has consistently demonstrated correlations between cognitive deficits and eye movement impairments in PD (Amador et al., 2006; Li et al., 2024a). For instance, Ba et al. (2022) revealed significant deficits in stereopsis, longer response times in gaze-related tasks (e.g., fixation stability and visual attention shifts), and reduced accuracy in saccades and fixations, highlighting the close association between these visual impairments and motor and cognitive dysfunctions. These findings suggest that stereopsis, measured using non-invasive tools like the 3D active shutter system and Tobii eye tracker, could be a useful marker for motor and cognitive function in PD. However, despite their clinical relevance, these tests are not widely adopted in routine practice. While advanced technologies such as shutter glasses and high-cost eye trackers may offer improved precision, their widespread adoption remains limited due to cost considerations and the variability of computer-based stereo testing algorithms. Further research is needed to evaluate their clinical efficacy compared to conventional methods and determine whether their benefits justify their financial and practical implementation in clinical settings.

Research suggests that reading difficulties are primarily linked to cognitive dysfunction rather than oculomotor deficits (Tsitsi et al., 2023). Prolonged fixation durations and reduced fixation frequency indicate impairments in executive function and working memory. Increased antisaccade errors and reduced saccade latencies correlate with executive dysfunction, while prolonged saccade latencies and hypometria are associated with broader cognitive decline. These findings highlight the potential of eye-tracking in assessing cognitive status and monitoring disease progression in PD (Waldthaler et al., 2023).

Moreover, eye-tracking offers insights into cognitive performance. The MoCA, a cognitive screening tool that includes executive function testing (Chou et al., 2014), has been shown to be more effective than the Mini-Mental State Examination (MMSE) in assessing cognitive impairments in PD, particularly in relation to eye movement behaviors (Tsitsi et al., 2021). Eye-tracking metrics, such as fixation duration and pupil size, have been correlated with cognitive performance, suggesting a connection between autonomic nervous system dysfunction and cognitive decline in PD.

Eye-tracking studies have also explored the influence of PD medications on oculomotor metrics. While nabilone showed no significant effects on saccadic paradigms, fixation, or top-down inhibitory control, a learning effect was observed, suggesting it does not impair cognitive consolidation (Ellmerer et al., 2022). Other studies (Birket-Smith, 1975; Roy-Byrne et al., 1993) have shown that medications like levodopa and anticholinergics affect pupil size and eye movement behaviors, particularly in patients with PDD (Brien et al., 2023). The influence of comorbidities and medications, such as antidepressants and benzodiazepines, on pupil size further underscores the need to consider these factors when interpreting eye-tracking data (Tsitsi et al., 2021).

Moreover, recent findings indicate that anti-Parkinsonian medication does not improve and may even worsen visually-guided saccades (Munoz et al., 2022). These results suggest that oculomotor impairments in PD are not solely dependent on dopaminergic dysfunction, but rather involve other neural pathways, such as cholinergic and fronto-striatal circuits. This aligns with evidence showing that saccadic impairments persist in both ON and OFF states, highlighting the complex neurophysiological mechanisms underlying oculomotor dysfunctions in PD.

Furthermore, eye-tracking has demonstrated value in non-motor assessments, such as facial emotion recognition. However, there is no consensus in the literature, as some studies have shown that PD patients struggle to recognize dynamic facial expressions, likely due to reduced facial expressiveness, which impairs their ability to use motion cues for emotion recognition (Brien et al., 2023). Research highlights the importance of task demands in shaping oculomotor behavior in PD. Beylergil et al. (2022) found no significant differences in saccade amplitudes between PD patients and controls during visual search tasks, aligning with previous research (Archibald et al., 2013). However, these results contrast with studies requiring memorization while scanning (Matsumoto et al., 2011), where PD patients exhibited smaller saccades and longer fixations. This suggests that tasks emphasizing active searching promote larger saccades and shorter fixations, whereas those involving memory recall result in more restricted eye movements.

Mobile eye-tracking can be an effective, non-invasive, and easy-to-use tool for clinical diagnosis, particularly in cases where traditional clinical criteria are ambiguous. Previous eye-tracking studies have primarily assessed visual activity in controlled, static laboratory environments (Backhaus et al., 2020). However, the development of mobile eye-tracking devices has recently enabled researchers to examine the effects of both PD and aging on visual exploration during real-world tasks, such as walking and navigating obstacle (Galna et al., 2012; Stuart et al., 2014; Stuart et al., 2016). Graham et al. (2023) explored visual search patterns while walking in PD patients with and without FOG using mobile eye-tracking and inertial sensors. The study found that FOG patients exhibited distinct gaze behaviors, including fewer fixations and reduced gaze variability, which correlated with impaired gait parameters. These findings highlight the role of eye-tracking in identifying visual exploration deficits linked to mobility issues in PD and suggest that gaze-based interventions could enhance gait performance. Although wearable eye-tracking has recently been proposed as a tool for various oculomotor and vestibular disorders (Hayhoe and Ballard, 2005; Schumann et al., 2008), it has been shown that wearable eye-tracking can also be effectively used in clinical settings for more complex conditions, such as typical and atypical Parkinsonism (Marx et al., 2012). Whether wearable eye-tracking will surpass the current diagnostic standards can only be determined through a long-term, prospective longitudinal study, which would apply the criteria identified here in the early stages of the disease, before clinical symptoms become fully evident.

Eye-tracking technology is also valuable for understanding visuospatial memory and eye movement dynamics in PD. For instance, in line with the literature (Hardeman et al., 2020; Ranchet et al., 2020; Sisk et al., 2018; Smith et al., 2021), a study by ŞtefŞnescu et al. (2024) found a moderate positive correlation between visuospatial memory performance and vertical eye movements, suggesting shared neural mechanisms involving the prefrontal-basal ganglia circuits. The study also discovered that blink rate, which is often reduced in PD patients, increases during cognitively demanding tasks, reflecting the impact of cognitive load on eye movement metrics. This variability in blink rate, along with its connection to working memory performance, indicates that eye-tracking can provide nuanced insights into cognitive and motor processes affected by PD.

### 4.2 Advances in eye-tracking technology: integrating machine learning and virtual reality for enhanced precision

Recent technological advancements are helping to overcome the limitations of traditional methods used to investigate eye abnormalities, such as spatial constraints and the inability to automatically diagnose PD. VR has emerged as a promising tool in this context, enabling more accurate evaluation and treatment of neurological and psychological cognitive disorders with greater assessment accuracy compared to conventional methods (Ferraioli et al., 2024; Karakoc et al., 2025; Lucifora et al., 2024; Nucera, 2024; Oliveira et al., 2018; Rizzo and Shilling, 2017; Vicario and Martino, 2022). However, despite its potential, few studies have examined eye movements within VR environments, and only one tool currently exists that can automatically classify PD (Jiang et al., 2024). In VR settings, three main types of eye movement data are collected: fixations, saccades, and synthetic features such as scan path length and duration. Studies have shown that PD patients exhibit significantly reduced saccade amplitude compared to HCs (Matsumoto et al., 2011). This leads PD patients to perform multiple corrective saccades to reach target locations, a behavior that worsens as the disease progresses. This abnormal eye movement pattern may also explain the mild visuospatial neglect often observed in PD, likely due to a restricted visual scanning area, which can contribute to issues such as dyslexia (Riva, 1997). Additionally, the saccade error rate (ER) was found to be significantly higher in PD patients, especially in tasks like Whack-a-Mole, indicating impaired inhibitory control of visually-guided saccades (VGS). This impairment is likely linked to dysfunctions in the cortical-basal ganglia-superior colliculus pathway, dopamine depletion in the prefrontal cortex, and the cognitive impairments associated with PD (Kavcic and Duffy, 2003).

Through tasks like VGS, it is possible to gain objective insights into cognitive control, helping to identify specific cognitive processes affected by PD and aiding in its diagnosis through distinct eye movement abnormalities. In parallel, the integration of ML has proven highly effective in developing automated, doctor-independent solutions for diagnosing and monitoring PD. Reflexive saccade (RS) data, for example, have been used to train intelligent classifiers, achieving over 90% accuracy in predicting PD-related features, making RS promising biomarker in PD diagnosis (Przybyszewski et al., 2014; Przybyszewski et al., 2016). VR-based eye-tracking, combined with ML, has proven to be a powerful tool for PD diagnosis and monitoring, as demonstrated by Jang et al. (2025), whose study showed that analyzing gaze stability, saccadic performance, and smooth pursuit in a VR setting, enhanced by deep learning models, achieved high diagnostic accuracy. Literature demonstrated that ML models based on eye-tracking data could efficiently assist neurologists in both diagnosing PD and monitoring the progression of symptoms (Szymañski et al., 2017). Specifically, RS measurements were crucial in building these classifiers, highlighting the importance of fast eye movements in detecting PD-related attributes. Further, the study suggested that systems like Eye Tribe, despite being lower-cost alternatives, could be effectively integrated into clinical settings to support PD diagnostics. Beyond RS, a variety of eye-tracking metrics, including those related to pro/anti saccade tasks, show alterations across different stages of PD progression. Recent studies have identified disturbances in pupil dilation and blinking as early markers during the prodromal stages of PD (Chambers and Prescott, 2010; La Morgia et al., 2022; Perkins et al., 2021; Waldthaler et al., 2021). These alterations in ocular behavior are now being further explored using ML techniques. Brien et al. (2023) developed a classifier that was sensitive to different stages of cognitive impairment in PD, from cognitively normal (PD-CN) through mild cognitive impairment (PD-MCI) to PDD.

The classifier demonstrated high accuracy, with eye movement measures correlating with clinical metrics such as the MDS-UPDRS and MoCA, suggesting that these metrics can effectively track disease progression. This reinforces the potential of eye-tracking metrics to capture the intricate relationship between cognitive and motor impairments in PD (Srulijes et al., 2015; Stuart et al., 2019; Waldthaler et al., 2021). Through ML models, this multidimensional data can be distilled into a single index predictive of PD subgroups and disease severity based on MDS-UPDRS scales. Consistent with earlier findings (MacAskill et al., 2012; Stuart et al., 2019), the output of these classifiers has shown sensitivity to both motor and cognitive functions, reinforcing the value of combining eye-tracking with ML in personalized treatment strategies. The use of eye-tracking combined with ML and VR technologies represents a significant advancement in PD diagnostics and monitoring. These tools offer a more precise, non-invasive, and automated approach to detect both motor and cognitive impairments in PD, promising better patient outcomes through early diagnosis and more personalized treatment strategies. The convergence of ET with ML and VR offers promising advancements in diagnostic and monitoring capabilities. Eye tracking data, when processed through ML algorithms, can help detect subtle ocular movement abnormalities associated with neurological disorders, enhancing early diagnosis and disease progression monitoring. VR, in combination with eye tracking, has been explored for cognitive and motor assessment in neurodegenerative diseases, providing an immersive and controlled environment for clinical evaluations (Jiang et al., 2025). However, integrating these technologies into existing clinical workflows remains complex. Healthcare systems must address issues related to data standardization and the development of AI-assisted decision-support tools that can provide meaningful insights to clinicians.

Although eye-tracking holds great promise for both research and clinical applications, its widespread use has been limited by the high cost and scalability issues related to specialized hardware. By leveraging the embedded cameras in mobile devices, these barriers can be overcome, making eye-tracking assessment tools more accessible (Valliappan et al., 2020). Tablet-based tools show potential for monitoring disease progression by assessing oculomotor function, as studies have demonstrated strong correlations between eye-movement parameters and clinical status. These tools could enable clinicians to remotely track changes in disease status, progression, or treatment response without the need for in-person visits, similar to approaches using wearable technologies like gyroscopes (Rodríguez-Molinero et al., 2018; Tripoliti et al., 2013) or speech analysis through ML. Eye-movement-based technologies offer the advantage of easier scalability to other neurodegenerative diseases, as several eye-movement anomalies have been linked to conditions like Alzheimer’s (Garbutt et al., 2008; Shakespeare et al., 2015) and multiple sclerosis (Lizak et al., 2016; Serra et al., 2018) with strong correlations to cognitive and clinical disease measure (Noiret et al., 2018; Waldthaler et al., 2019).

While eye-tracking technology has demonstrated significant potential in clinical applications, its integration into hospital and healthcare systems remains a challenge. Successful adoption requires substantial investment in infrastructure, including specialized hardware, software integration, and compatibility with existing electronic health record systems. Moreover, healthcare institutions must ensure the availability of technical support and standardized protocols to facilitate seamless implementation. Literature highlights the need for dedicated resources to optimize eye-tracking data collection and analysis in clinical environments. While some research institutions and specialized clinics have begun incorporating eye-tracking into neurology assessments, large-scale integration into routine clinical practice is still in progress. Key barriers include the lack of standardized protocols, the need for regulatory approval, and limited awareness among medical professionals regarding eye-tracking’s potential benefits. Surveys suggest that while clinicians acknowledge eye-tracking’s promise, further validation studies and practical guidelines are necessary to promote broader acceptance. Overcoming these challenges will be crucial to establishing eye-tracking as a reliable and scalable tool in neurology (Gibbs et al., 2024).

In conclusion, eye-tracking presents significant potential as a non-invasive, precise tool for diagnosing and monitoring PD. It not only captures motor impairments but also provides valuable insights into cognitive dysfunctions (Pinkhardt et al., 2009). By assessing pupil responses, saccades, or blink rates, eye-tracking can contribute to a more comprehensive understanding of PD progression (Gorges et al., 2017). As wearable and mobile eye-tracking systems continue to evolve, they could be integrated into routine clinical practice to enhance diagnostic accuracy and provide personalized treatment strategies, particularly in cases where traditional criteria are ambiguous or insufficient. This review offers a novel perspective by integrating recent advancements in eye-tracking technology, including mobile and wearable eye tracking, VR-based assessments, and ML models for PD diagnosis and symptom monitoring. It synthesizes findings on the clinical feasibility and real-world applications of eye tracking, emphasizing its potential for routine implementation in clinical practice.

Future studies should focus on including prodromal PD populations, where early motor and non-motor symptoms are present, but a formal diagnosis has not yet been established. These individuals may exhibit subtle eye movement abnormalities that could serve as early indicators of disease onset. While recruiting asymptomatic participants and prospectively monitoring their conversion to PD poses practical challenges, recent advances in biomarker research, such as the α-syn seed amplification assay, may enable the identification of high-risk individuals (Yamasaki et al., 2019). This could provide a unique opportunity to explore the diagnostic potential of eye-tracking in the earliest stages of PD, before traditional clinical symptoms become apparent.

To enhance clarity in future research, it is important to distinguish between the different applications of eye-tracking in PD. Eye-tracking can be used for diagnosing PD, which involves identifying the disease in individuals without a prior diagnosis; classifying PD, which refers to distinguishing patients with PD from HCs or other neurodegenerative conditions; and monitoring PD, which entails assessing disease progression over time. Standardizing these definitions across studies will help ensure methodological consistency and improve the comparability of findings in the field.

### 4.3 Opportunities and challenges for eye-tracking studies

The eye-tracking technique offers several advantages, such as being well-tolerated due to the short duration of tasks, like the 5-min visual search task (Wong et al., 2019). Additionally, one notable strength of wearable eye-tracking devices is their efficiency, requiring less than 20 s for fixation protocols and virtually no device-specific training, making them practical for clinical and experimental use (Marx et al., 2012). However, clinicians should carefully consider the most appropriate approach when selecting an eye tracker, as some devices require head stabilization using a chin rest, while others allow for unsupported head movement. Many eye trackers are stationary, but some are portable, and others, such as the Tobii Pro Glasses, are mobile and wearable, enabling participants to engage in everyday tasks (de Villers-Sidani et al., 2023; Jiang et al., 2024). However, systems with variable frame rates, like the Tobii Pro Glasses, may be unsuitable for precise saccade analysis due to potential inconsistencies in data capture. This limitation should be considered when selecting an eye-tracking system for research involving rapid eye movements. Selecting the right device depends on the specific needs of the clinical evaluation. Although various studies used different eye-tracking devices, it is challenging to determine which one is superior.

There are several limitations to eye-tracking research in PD. For instance, malfunctions can occur with participants wearing progressive lenses, and individuals with conditions such as eyelid apraxia or certain ophthalmological disorders may find the device difficult to use. While eye-tracking serves as a physiological marker that is largely unaffected by pre-existing conditions or intelligence, many studies have not provided information on participants’ premorbid intelligence, making it difficult to assess the true extent of cognitive decline (Wong et al., 2019). Additionally, the absence of neuroradiological data in many studies means that undetected intracranial pathologies could influence both cognitive performance and eye movement measurements (Wong et al., 2019). Another factor is the relatively younger PD cohorts used in some studies, where the decline in cognition and eye movements may differ from those in older populations. The heterogeneity of PD and the exclusion of participants in the more advanced stages (Hoehn and Yahr stages 4 and 5) (Brien et al., 2023) limit the generalizability of findings, as cognitive decline and eye movement abnormalities may differ significantly in these groups. Moreover, general considerations related to behavioral experiments with older and neurologically-impaired participants should be noted, such as the potential impact of fatigue or discomfort on attention and engagement during prolonged testing sessions. The small sample sizes in some studies also present a challenge, limiting the robustness of the findings (Ba et al., 2022; Stuart et al., 2016). It is important to acknowledge that all studies included in this review enrolled PD patients after-diagnosis, indicating that eye-tracking metrics were primarily analyzed in individuals at a disease stage likely beyond the earliest clinical manifestation. This presents an important limitation, as the generalizability of these oculomotor biomarkers to prodromal or very early PD remains uncertain. In earlier disease stages, when symptom expression is more subtle, the signal-to-noise ratio in eye movement abnormalities may be weaker, potentially affecting the sensitivity of eye tracking-based classification methods. Furthermore, while many reviewed studies demonstrated the ability of eye-tracking to distinguish PD patients from HCs, fewer have focused on actual diagnostic applications and its reliability for individual diagnosis remains under investigation. Validating these findings in larger, longitudinal cohorts, including prodromal PD patients, to better determine the clinical utility of eye-tracking for early disease detection and monitoring. Additionally, future research should consider the potential impact of medications, sex differences, and other confounding factors, as these variables have not been thoroughly investigated. In terms of future directions, improving study designs by increasing sample sizes, optimizing tasks and equipment, and addressing technical challenges would strengthen the validity of eye-tracking studies.

## 5 Conclusion

This systematic review highlights the increasing value of eye-tracking as a non-invasive, objective tool for diagnosing and monitoring PD. Eye-tracking technology enables precise measurement of oculomotor functions that correlate with both motor and cognitive symptoms of PD, potentially providing early diagnostic markers and facilitating the monitoring of disease progression. Technological advancements, including the integration of ML and VR, have expanded the diagnostic potential of eye-tracking. Our review builds upon previous literature (e.g., Waldthaler et al., 2021) by focusing on the latest methodological and technological innovations, and highlights emerging opportunities for more precise, scalable, and automated assessments of PD through eye-tracking.

However, challenges persist, such as device variability, limitations in cognitive assessment, and the need for larger, more diverse sample sizes. Future research should focus on standardizing eye-tracking protocols and further exploring its application across different neurodegenerative disorders to enhance diagnostic accuracy and improve patient outcomes. In this regard, the International Society for Clinical Eye tracking^1^ is currently working on recommendations for standardized testing protocols in clinical applications. Additionally, integrating eye-tracking into routine clinical practice could provide better personalized treatment strategies, particularly in cases where traditional clinical criteria are insufficient.

## Data Availability

The data analyzed in this study is subject to the following licenses/restrictions: the data that support the findings of this article are available on request from the corresponding author. Requests to access these datasets should be directed to DC, davide.cardile@irccsme.it.

## Footnotes

1
https://clinicaleyetracking.org/
